# Impact of COVID-19 on Blood Donation and Supply: A Multicenter Cross-Sectional Study from Saudi Arabia

**DOI:** 10.1155/2022/1474426

**Published:** 2022-01-12

**Authors:** Nora Y. Hakami, Afnan J. Al-Sulami, Wafaa A. Alhazmi, Talal H. Qadah, Waleed M. Bawazir, Abdullah Y. Hamadi, Amani Y. Owaidah, Razan A. Alhefzi, Fawaz Y. Hamdi, Amr Maqnas, Ghassab F. Alghassab, Maha A. Badawi, Salwa I. Hindawi

**Affiliations:** ^1^Department of Medical Laboratory Technology, Faculty of Applied Medical Sciences, King Abdulaziz University, Jeddah, Saudi Arabia; ^2^Blood Transfusion Services, King Abdulaziz University Hospital, Jeddah, Saudi Arabia; ^3^Hematology Research Unit, King Fahd Medical Research Center, King Abdulaziz University, Jeddah, Saudi Arabia; ^4^Department of Medical Laboratory Technology, Faculty of Applied Medical Sciences, University of Tabuk, Tabuk, Saudi Arabia; ^5^Department of Clinical Laboratory Sciences, Collage of Applied Medical Sciences, Imam Abdulrahman Bin Faisal University, Dammam, Saudi Arabia; ^6^Department of Clinical Laboratory Sciences, Faculty of Applied Medical Sciences, King Khalid University, Abha, Saudi Arabia; ^7^Department of Clinical Laboratory, Samtah General Hospital, Ministry of Health, Samtah, Saudi Arabia; ^8^Saudi Food and Drug Authority, Riyadh, Saudi Arabia; ^9^Regional Blood Bank, Hail, Saudi Arabia; ^10^Department of Hematology, Faculty of Medicine, King Abdulaziz University, Jeddah, Saudi Arabia

## Abstract

**Background:**

The coronavirus disease-19 (COVID-19) pandemic caused a major impact on blood donation process and supply globally. A lockdown management procedure was launched nationally in Saudi Arabia to manage this global health crisis. The main aim of this study was to determine the effect of COVID-19 lockdown on blood donation services and supply in different regions of Saudi Arabia. *Study Design and Methods*. A retrospective cross-sectional study was conducted in the blood bank centers of 5 major cities including Riyadh, Jeddah, Dammam, Hail, and Jizan in Saudi Arabia. Demographic and blood characteristics were retrieved from the first 6 months of 2019 (January–June) and compared to the same period of 2020.

**Results:**

Our findings showed variation in the characteristics of blood donation and supply among the centers surveyed, as some of these centers were adversely affected, while others showed an increase in the availability of blood products during the pandemic. For example, Jeddah's center was significantly affected by COVID-19 lockdown whereas Hail's center showed a significant increase in the analyzed characteristics of blood donation services in 2020 compared to 2019. Overall, there was no major difference among the surveyed centers between 2020 and 2019, and this might be due to the effective management of blood supply and transfusion. *Discussion*. Although blood supply and transfusion practice was slightly affected at various degree among the surveyed centers, the whole process did not show a significant effect on the overall outcome. This is in fact due to the proper preparedness, management of blood requirements and supplies, and efficient response of the surveyed centers in Saudi Arabia.

## 1. Introduction

Blood donation remains the main source of blood products consumption [1]. It is a vital process to save lives of the needed patients who have anemia, serious injuries, surgical operations, and chronic disease complications [2]. Coronavirus disease 2019 (COVID-19) was announced as a pandemic infection by the World Health Organization (WHO) last year resulting in a public health emergency situation [3]. Severe disruption to both supply and demand for blood donation and products are among the implications caused by COVID-19 pandemic. Blood donation was dropped by 40% to 67% during the COVID-19 outbreak, especially in regions where movement restrictions were in place [4]. It has been revealed that 13.4% of COVID-19 patients who were admitted to the hospital required blood transfusions, which was lower than the number of non-COVID-19 patients. Furthermore, the red blood cells (RBCs) were reported as the most frequently transfused blood product (11.1%) [5]. Although most of the countries took strict precautionary measures to control the infection, COVID-19 still threatens human life specially with reporting emerged mutated strains and the second wave of infection. In addition to maintain the spread of COVID-19, the healthcare centers and hospitals are still working to provide emergency facilities for patients suffering from other medical conditions. The continuous supply of a safe blood for transfusion is vital for all these cases [5]. Previous experience with other viral outbreaks such as influenza and coronaviruses showed a low risk of transfusion transmission; however, the safety measures aligned with the infection control noticeably affected the blood donation services [6–8]. During the epidemic of severe acute respiratory syndrome (SARS) reported in China at 2003, Beijing's blood supply was significantly disturbed due to movement constriction caused by avoidance of people to be out in the public areas and the panic of catching the virus at the centers for donation. This resulted in calling for a help request from centers located in less affected areas of the world [7, 9]. During the H1N1 pandemic, a study performed in Japan revealed a rapid decrease in donor numbers by 21% within one week of the first reported case resulting in a strong mobilization of donors [10]. During the current COVID-19 pandemic, the virus itself did not cause a direct impact on blood supply, but rather, the unexpected effect of social distancing on blood donation and blood products may contribute to this impact [11]. This had led to a large reduction in the number of stock inventories and regional donation centers. In several countries, the pattern of decline in voluntary donations was globally well observed despite the tremendous efforts made by health authorities and the media to assure safe practices and precautions to prevent the transmission of infection in the blood donation areas [1, 11–13]. The WHO has given a preliminary guidance to maintain a safe and sufficient blood supply during the COVID-19 pandemic [14]. The European Center for Disease Prevention and Control (ECDC) [15] and the American Association of Blood Banks (AABB) [16] presented fast risk assessments of the SARS-CoV-2 outbreak and blood safety in January 2020. Following the strategy adopted for SARS-CoV and MERS-CoV, the ECDC recommended a 21-day moratorium on blood and cell donations after possible exposure to a confirmed COVID-19 case or anyone who returned from Wuhan, China. Additionally, recovering COVID-19 patients should be postponed for at least 28 days after symptom remission and treatment completion [15]. The AABB had updated its website to state that they would continue to closely monitor the respiratory illness outbreak due to concerns about SARS-CoV-2 and blood safety. The AABB, the Food and Drug Administration, and the Centers for Disease Control and Prevention do not currently require any further action on blood collection and testing as there is no reported evidence of SARS-CoV-2 transmission by transfusion [9]. There is a need for transfusion medicine experts to play an active role in crisis coordination and provide data to the authorities to discuss the feasibility of their safety guidelines. Shared expertise and policies across regional transfusion networks and foreign organizations have been found to be beneficial during infection outbreak to maintain access to the right advice and procedures during the period of such crisis [6]. However, the background of the effect of the COVID-19 pandemic on blood supply in local area around Saudi Arabia is not yet fully covered, and more information is required. Therefore, the main aim of this study is to determine the impact of COVID-19 on blood donation management before and during the COVID-19 pandemic in different cities of Saudi Arabia.

## 2. Materials and Methods

### 2.1. Study Design and Data Collection

A cross-sectional study was undertaken in 5 centers in the main cities representing different provinces of Saudi Arabia during the first six months (January–June) of 2019 and 2020. These cities included Riyadh, Jeddah, Dammam, Hail, and Jizan. Demographic data included nationality, gender, and number of total and rejected donors. Other measured parameters included the average numbers of different blood products such as packed red blood cell (PRBC) bags, number of fresh frozen plasma (FFP), number of platelet concentration (Plt conc.), number of cryoprecipitates (Cryo), and number of apheresis. Donors participating in this study meet all donation criteria and requirements. In addition, they fulfilled the following inclusion criteria: no travel history within the past 28 days, no contact with someone with suspected or confirmed COVID-19 infection within the past 28 days, and donors who are neither suspected nor confirmed to have COVID-19 infection within the past 28 days.

### 2.2. Ethical Approval

This study was approved by the bioethics and research committee of King Abdulaziz University Hospital, reference no. 499-20 dated, September 30, 2020, and the ethical committee of Ministry of Health, IRB registration no. H-02-J-002. Recruited participants were informed of using their clinical data for clinical and research purposes with the confidentiality of all personal data.

### 2.3. Data Analysis

All results were expressed as mean +/- standard deviation and analyzed by IBM SPSS Statistics for Windows, version 23 (IBM SPSS, IBM Corp., Armonk, N.Y., USA). Shapiro–Wilk test was used to evaluate normal data distribution. Student *t*-test was used to calculate significance between parametric variables of normally distributed data, while Mann–Whitney test was used for nonparametric data. *p* values < 0.05 were considered statistically significant.

## 3. Results

Comparison of blood donors in the 1st six months of the years 2019 and 2020 was conducted in major cities of Saudi Arabia. Our results showed variation in the characteristics of blood donation and supply among the studied centers in the five major cities. Some of these centers were adversely affected while others showed an increase in the availability of blood products during the pandemic lockdown. The data obtained by this study reject the null hypothesis that expects no difference or effect and confirm an alternative hypothesis that some differences or effects were found between cities.

Our results showed that the center in Jeddah was the most affected one during the pandemic as most of the characteristics showed a significant drop in 2020 compared to 2019 (*p* < 0.05). These results were the total number of donors (*p* = 0.02) including Saudi (*p* = 0.033) or non-Saudi donors (*p* = 0.018), male donors (*p* = 0.011), the number of PRBCs (*p* = 0.022), FFP (*p* = 0.020), and platelet concentrates (*p* = 0.007) as shown in (Table 1). However, the number of recruited female donors, cryoprecipitate and apheresis did not show any significance difference.

This was followed by Riyadh (Table 2) and Dammam (Table 3) centers where three characteristics were distinctively affected, but only one was significantly increased (*p* < 0.05). Both Riyadh and Dammam centers were significantly affected in the number of rejected donors (*p* = 0.014 and 0.015) and the number of platelet concentrates (*p* = 0.0001 and 0.018), respectively. Nevertheless, both cities revealed a significant increase in the number of apheresis (*p* = 0.008 and 0.011), respectively, during 2020 compared to 2019. The number of FFP was significantly dropped in Riyadh (*p* = 0.049) but not in Dammam while the number of cryoprecipitate was reported to be declined in Dammam (*p* = 0.026) but not in Riyadh.

In Jizan center, the impact of COVID-19 pandemic showed a mild effect on blood donation services and transfusion (Table 4). A significant drop was observed in the number of non-Saudi donors only (*p* = 0.003) while other characteristics were insignificantly decreased. It is noteworthy to mention that we were not able to trace the number of cryoprecipitate and apheresis due to the unavailability of data owing to technical difficulties.

Conversely and interestingly, in Hail, there was a significant increase in all characteristics of blood donation except for the number of cryoprecipitate (Table 5) indicating an effective management of blood donation services during the pandemic period. The significant increase (*p* < 0.05) was observed in the number of recruited donors including Saudi and non-Saudi male or female donors, the number of rejected donors, and the number of PRBCs, FFP, platelet concentrates, and apheresis. However, a significant drop was only notable in the number of cryoprecipitate (*p* = 016).

Collectively, all the characteristics of blood donation among centers in all studied cities showed no major changes (*p* > 0.05) between 2019 and 2020 despite the reduction in all parameters except for female donors (Table 6). This reduction was observed in the total number of donors, including Saudi and non-Saudi male donors, rejected donors, and the number of PRBCs, FFP, platelet concentrates, and apheresis.

## 4. Discussion

Blood donation and transfusion play an important role in medicine and public health. Blood and its components are an integral part of standard clinical practice, delivering life-saving treatments and benefits to patients by dramatically increasing their average lifespan and performance in both acute and chronic conditions [17]. It is critical to emphasize the importance of the blood supply for first aid, surgery, the treatment of certain disorders (e.g., oncological diseases), transplants, and transfusions. As a result, self-sufficiency of blood supply is unquestionably essential for both regional hospitals and healthcare centers as there are high demands for blood donation [18]. The government rewards voluntary blood donors with a vacation, a stipend, and priority access to blood transfusions during emergencies to encourage blood donation [19]. To overcompensate for a 10% drop in blood donations in the first week of March, the government launched a national media campaign emphasizing the necessity and safety of blood donation as a priority for maintaining non-COVID patients' essential healthcare services [20].

Data from previous studies also showed that infectious outbreaks are likely to affect the blood supply and the use of blood products [6, 7, 21, 22]. For example, a previous report presented that the pandemic of influenza H1N1 virus had a significant impact on blood supply and management [6]. Another example, the emergence of the first SARS epidemic in China had also resulted in a disruption of the blood management system and negatively affected the availability of blood components [7].

**Figure 1:**
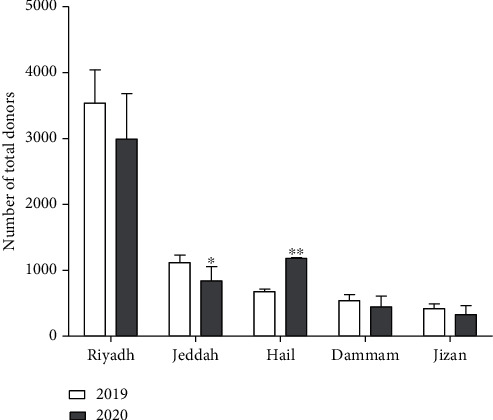
Number of total donors in different regions of Saudi Arabia in period from January to June 2019 and 2020. (^∗^) indicates the significance of *p* < 0.05; (^∗∗^) indicated the significance of *p* < 0.005.

Our work indicated that COVID-19 pandemic had affected most Saudi cities at variable degrees of detriment on blood donation and transfusion processes. The blood bank center of Jeddah city was severely affected by the pandemic compared to the blood bank centers from Riyadh, Jizan, and Dammam that were slightly affected (Figure 1). This variability in the decrease of blood donation and supply characteristics among the studied centers occurred due to several reasons such as people awareness, management of donor recruitment campaigns, movement restriction, and lockdown measures. This is evidenced by a number of previous and recent studies performed in other parts of the world such as China, Italy, and USA [11, 23, 24]. A recent study has investigated the impact of COVID-19 pandemic on blood donation and supply in Zhejiang province, China, through self-administered questionnaire from 38 blood centers. It was found that the number of blood donors decreased by 67% and the number of RBCs supply dropped by 65%. These results were due to the lack number of donors but not the COVID-19 pandemic itself. The same study reported that the majority of people were anxious from catching the virus during the donation process and thus not willing to donate [11]. A very recent study conducted in the Eastern Mediterranean Region has evaluated the negative impact of the COVID-19 pandemic in the first five months. This study showed that there was a distinct reduction in the blood supply and donations in most centers due to the public fear from the pandemic [25]. Moreover, a recent report performed in Italy found that a reduction by 10% in blood donations every week was noticed due to movement constrictions during the COVID-19 lockdown. However, blood inventory was kept under control due to postponement of elective surgeries [23]. Moreover, a study by Yahia has recently published their experience with blood supply and demand in Bisha, Saudi Arabia. There was a substantial decrease in the blood bank-based collections by 39.5% as well as a decrease in blood demand by 21.7% at the same time [1]. This was explained and clarified by different studies that multiple factors could negatively influence the number of blood donors during a viral pandemic. For example, owing to closures, common outlets of voluntary blood donations such as hospitals were affected. External influences such as social distancing and lockdown can hinder the accessibility to blood donation centers. Donors may also be hesitant to visit the blood bank due to the fear of being infected, and organizations may be unwilling to hold charity events to avoid the transmission of the virus to the population [6].

Since our results showed no distinct change between 2019 and 2020 when looking at all cities collectively, this would mean an appropriate blood donation service management was applied by the health authorities in terms of handling logistic issues and ensuring the implementation of infection control and prevention measures. Our finding was supported by a recent study conducted at a tertiary care hospital in Western India which observed the blood transfusion inventory before and during the COVID-19 lockdown periods (Ref). During the pandemic, postponement of elective surgeries was among the effective strategies for blood supply which may explain the insignificant change in our study [26]. This was also evidenced by a recent study reported a decline in the demand for blood units due to the deferral of elective surgeries and regular procedures during the COVID-19 pandemic. The latter study showed that the need for PRBC, fresh frozen plasma, and platelets was reduced by 14%, 11%, and 1.6%, respectively [4]. This finding could explain similar result in our study conclusion.

Interestingly, we reported that Hail city located in the northern province was the only city found with a significant increase in the number of blood donors in 2020 compared to 2019. Altruism has been reported to be the major reason for keeping donors to be motivated. In addition, religious act, national duty, quality of service, and family encouragement may play roles to some extent in motivation for blood donation [27]. Before giving blood products, a blood donor should meet several Federal Drug Administration (FDA) standards. Physical standards including age, weight, temperature, blood pressure, and pulse are among them, as well as the background check of the donor's sexual, medical, and travel histories. Any contradictions or difficulties occurred during the interview, and physical examination could cause the donor to be removed from the blood donation system, either temporarily or permanently [28].

Since our overall results showed no significant effect, this may reflect an effective management and strategy action taken by the health authorities to ensure blood donors' and workers' protection safety during blood donation. Among these actions, an extra blood donor screening questionnaire was developed to assess donors with an elevated risk of COVID-19 exposure [29]. Other precautionary measures include deferment of donors returning within 14 days from any national or international territory, deferment of donors who have been in close contact with confirmed cases or showing symptoms of COVID-19, and donors with a body temperature more than 37.5°C [30, 31]. The FDA published a new blood donation guidance on April 2, 2020, to meet the need for blood and blood components. They removed the requirement that collections would be rejected if vital signs or donation intervals were incorrect, instead they established a 72-hour opening for donors to answer queries regarding their eligibility and appropriateness [28].

In this study, the main strategic policies were updated and applied by the major Saudi donation centers during the pandemic to include the following criteria: check the temperature prior donation, asking donors if they experienced any symptoms of fever or cough close contact to affected cases, and reporting their recent travel history, availability of hand sanitizer and face masks, and arrangement of seats at 2-meter gap (keeping distance). According to ministry of health policy and regulation, the effective management to maintain the blood supply might be achieved through increasing the number of donation campaigns, organizing drives in military environments, and intensifying marketing and donor training, including mobilizing donors from health care personnel. In addition, keeping a hotline communication for public inquiries was among the most effective step to control the situation [14]. This is probably due to several factors related to effective handling and management of the pandemic as well as the awareness of blood donors [27, 32]. However, there are some limitations in this research. For example, the current study did not include all cities in Saudi Arabia. Furthermore, because this study was conducted early in the pandemic, the results obtained may differ later.

## 5. Conclusion

Although blood supply and transfusion practices were affected at various degree among the surveyed centers, the whole process did not show a major impact on the overall outcome. This is in fact due to proper preparedness, management of blood requirements and supplies, and efficient response of the surveyed centers in Saudi Arabia. Awareness and effective blood donation campaigns, patient blood management through rescheduling surgeries, minimizing the use of blood components, and applying the measures to reduce bleedings all have contributed to keep blood component inventory and supply within the needed level. However, there is a space for improvement plan, particularly to limited-shelf-life blood component such as platelets, to review donor's deferral period and developing donor questionnaire which are quite necessary. We emphasize on regular public awareness strategies, protection of blood donors, and the need to ensure a secure and appropriate blood supply from regular blood donors.

## Figures and Tables

**Table 1:** Comparison of blood transfusion characteristics of participants in Jeddah from January to June in years 2019 and 2020.

Characteristics	Jeddah		Significance
	2019	2020	
No. of total donors	1114.50 ± 114.91	839.17 ± 216.31	**0.020**
No. of Saudi donors	631.83 ± 64.15	479.50 ± 136.29	**0.033**
No. of non-Saudi donors	482.67 ± 54.85	359.83 ± 90.80	**0.018**
No. of rejected donors	181.50 ± 76.49	88.50 ± 63.62	**0.045**
No. of female donors	90.33 ± 32.19	76.83 ± 39.64	0.532
No. of male donors	1015.17 ± 102.69	747.83 ± 182.57	**0.011**
No. of PRBCs	1069.50 ± 112.29	806.33 ± 208.79	**0.022**
No. of FFP	422.17 ± 60.40	267.50 ± 123.09	**0.020**
No. of Plt conc.	783.33 ± 69.80	603.33 ± 110.31	**0.007**
No. of Cryo	44.50 ± 15.97	45.00 ± 24.19	0.967
No. of apheresis	9.50 ± 3.39	10.33 ± 4.13	0.711

Data were expressed as mean +/- standard deviation (SD). Significance between groups was calculated using Student *t*-test for parametric data and # Mann–Whitney test for nonparametric data.

**Table 2:** Comparison of blood transfusion characteristics of participants in Riyadh from January to June in years 2019 and 2020.

Characteristics	Riyadh		Significance
	2019	2020	
No. of total donors	3536.67 ± 503.94	2988.83 ± 689.07	0.147
No. of Saudi donors	1793.50 ± 339.86	1518.83 ± 447.15	0.259
No. of non-Saudi donors	1576.50 ± 367.16	1470.00 ± 285.96	0.587
No. of rejected donors	535.67 ± 70.43	309.50 ± 172.07	**0.014**
No. of female donors	89.67 ± 36.99	105.00 ± 40.54	0.509
No. of male donors	3446.83 ± 481.57	2881.67 ± 712.81	0.139
No. of PRBCs	3159.67 ± 420.57	2478.67 ± 644.40	0.055
No. of FFP	2754.67 ± 394.67	2037.67 ± 677.89	**0.049**
No. of Plt conc.	2577.00 ± 339.54	1117.33 ± 332.43	**0.0001**
No. of Cryo	158.00 ± 74.62	177.17 ± 85.90	0.689
No. of apheresis	83.17 ± 29.38	137.17 ± 27.34	**0.008**

Data were expressed as mean +/- standard deviation (SD). Significance between groups was calculated using Student *t*-test for parametric data and # Mann–Whitney test for nonparametric data.

**Table 3:** Comparison of blood transfusion characteristics of participants in Dammam from January to June in years 2019 and 2020.

Characteristics	Dammam		Significance
	2019	2020	
#No. of total donors	539.33 ± 91.82	443.50 ± 163.95	0.240
#No. of Saudi donors	523.67 ± 82.63	431.33 ± 159.49	0.394
#No. of non-Saudi donors	15.67 ± 17.71	12.17 ± 8.04	0.818
#No. of rejected donors	109.00 ± 57.44	33.67 ± 37.10	**0.015**
#No. of female donors	5.83 ± 1.60	7.67 ± 7.61	0.699
#No. of male donors	533.50 ± 90.93	435.83 ± 159.76	0.240
No. of PRBCs	343.33 ± 45.40	335.83 ± 136.92	0.901
No. of FFP	342.50 ± 44.72	336.67 ± 136.94	0.922
No. of Plt conc.	343.33 ± 45.65	337.33 ± 138.08	**0.018**
#No. of Cryo	43.00 ± 21.00	8.67 ± 21.23	**0.026**
No. of apheresis	1.83 ± 1.84	4.50 ± 1.64	**0.011**

Data were expressed as mean+/- standard deviation (SD). Significance between groups was calculated using Student *t*-test for parametric data and # Mann–Whitney test for nonparametric data.

**Table 4:** Comparison of blood transfusion characteristics of participants in Jizan from January to June in years 2019 and 2020.

Characteristics	Jizan		Significance
	2019	2020	
No. of total donors	419.83 ± 67.60	326.50 ± 137.78	0.167
No. of Saudi donors	319.83 ± 72.52	266.50 ± 114.68	0.358
No. of non-Saudi donors	100.00 ± 7.54	60.00 ± 24.63	**0.003**
No. of rejected donors	32.83 ± 7.99	30.83 ± 5.53	0.625
#No. of female donors	13.17 ± 4.71	7.67 ± 17.82	0.065
No. of male donors	406.67 ± 64.94	318.83 ± 127.27	0.163
No. of PRBCs	387.00 ± 61.81	295.67 ± 133.25	0.159
No. of FFP	387.00 ± 61.81	295.67 ± 133.25	0.159
#No. of Plt conc.	192.17 ± 18.86	157.17 ± 42.79	0.092
No. of Cryo	—	—	**—**
No. of apheresis	—	—	**—**

Data were expressed as mean +/- standard deviation (SD). Significance between groups was calculated using Student *t*-test for parametric data and # Mann–Whitney test for nonparametric data.

**Table 5:** Comparison of blood transfusion characteristics of participants in Hail from January to June in years 2019 and 2020.

Characteristics	Hail		Significance
	2019	2020	
#No. of total donors	677.50 ± 35.83	1179.17 ± 12.02	**0.002**
#No. of Saudi donors	646.67 ± 32.53	1137.83 ± 10.80	**0.002**
No. of non-Saudi donors	30.83 ± 3.49	41.33 ± 1.97	**0.0001**
No. of rejected donors	9.67 ± 1.21	17.83 ± 2.04	**0.0001**
No. of female donors	3.50 ± 1.87	14.33 ± 2.81	**0.0001**
#No. of male donors	673.83 ± 35.43	1164.83 ± 9.73	**0.002**
#No. of PRBCs	634.17 ± 9.87	1047.17 ± 18.98	**0.002**
#No. of FFP	634.17 ± 9.87	1047.17 ± 18.98	**0.002**
#No. of Plt conc.	634.17 ± 9.87	1047.17 ± 18.98	**0.002**
No. of Cryo	18.00 ± 4.98	11.33 ± 2.58	**0.016**
No. of apheresis	1.17 ± 2.04	5.50 ± 2.74	**0.011**

Data were expressed as mean +/- standard deviation (SD). Significance between groups was calculated using Student *t*-test for parametric data and # Mann–Whitney test for nonparametric data.

**Table 6:** The average of blood transfusion characteristics of all participants in all regions in Saudi Arabia from January to June in years 2019 and 2020.

Characteristics	All regions		Significance
	2019	2020	
No. of total donors	7545.40 ± 7805.13	6932.60 ± 6473.28	0.896
No. of Saudi donors	4698.60 ± 3478.35	4600.80 ± 3215.18	0.964
No. of non-Saudi donors	2846.80 ± 4406.22	2332.00 ± 3723.54	0.847
No. of rejected donors	1042.40 ± 1279.90	576.40 ± 734.12	0.500
No. of female donors	243.00 ± 271.97	253.80 ± 273.39	0.952
No. of male donors	7291.20 ± 7608.25	6658.80 ± 6259.46	0.889
No. of PRBCs	6712.40 ± 7060.73	5958.20 ± 5333.89	0.854
No. of FFP	5448.60 ± 6229.92	4781.60 ± 4594.46	0.852
No. of Plt conc.	5436.00 ± 5776.33	3914.80 ± 2543.70	0.605
No. of Cryo	316.20 ± 370.24	290.60 ± 443.83	0.924
No. of apheresis	115.00 ± 216.39	189.00 ± 355.10	0.701

Data were expressed as mean +/- standard deviation (SD). Significance between groups was calculated using Student *t*-test for parametric data and # Mann–Whitney test for nonparametric data.

## Data Availability

No data was used.

## Abbreviations

COVID-19: Coronavirus disease 2019
SARS-CoV-2: Acute respiratory syndrome coronavirus 2
PRBCs: Packed red blood cells
FFP: Fresh frozen plasma
Plt conc.: Platelet concentration
Cryo: Cryoprecipitates.
